# 4-(4-Fluoro­phen­yl)-1-phenyl-3-(pyridin-4-yl)-1*H*-pyrazol-5-amine

**DOI:** 10.1107/S1600536812004096

**Published:** 2012-02-10

**Authors:** Bassam Abu Thaher, Pierre Koch, Dieter Schollmeyer, Stefan Laufer

**Affiliations:** a Faculty of Science, Chemistry Department, Islamic University of Gaza, Gaza Strip, Palestinian Territories; b Institute of Pharmacy, Department of Pharmaceutical and Medicinal Chemistry, Eberhard Karls University Tübingen, Auf der Morgenstelle 8, 72076 Tübingen, Germany; c Department of Organic Chemistry, Johannes Gutenberg University Mainz, Duesbergweg 10-14, D-55099 Mainz, Germany

## Abstract

In the title compound, C_20_H_15_FN_4_, the pyrazole ring forms dihedral angles of 43.51 (6), 39.95 (6) and 32.23 (6)° with the directly attached 4-fluoro­phenyl, pyridine and phenyl rings, respectively. The crystal packing is stabilized by inter­molecular N—H⋯N and N—H⋯F hydrogen bonds.

## Related literature

For p38α MAP kinase inhibitors having a vicinal 4-fluoro­phen­yl/pyridin-4-yl system connected to a five-membered heterocyclic core, see: Abu Thaher *et al.* (2009 ▶); Peifer *et al.* (2006 ▶). For inhibitory activity and preparation of the title compound, see: Abu Thaher *et al.* (2012 ▶).
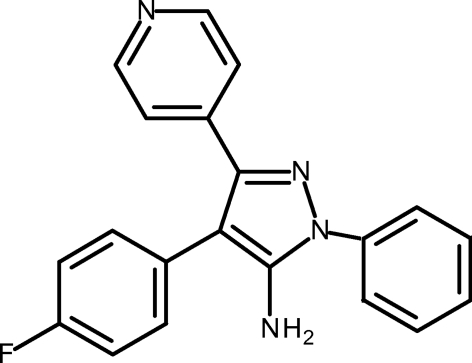


## Experimental

### 

#### Crystal data

C_20_H_15_FN_4_
*M* *_r_* = 330.36
Monoclinic, 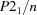

*a* = 12.2408 (3) Å
*b* = 10.4427 (2) Å
*c* = 12.9099 (3) Å
β = 101.951 (1)°
*V* = 1614.46 (6) Å^3^
*Z* = 4
Mo *K*α radiation
μ = 0.09 mm^−1^
*T* = 193 K
0.39 × 0.38 × 0.24 mm

#### Data collection

Bruker SMART CCD diffractometer
29688 measured reflections
5459 independent reflections
3972 reflections with *I* > 2σ(*I*)
*R*_int_ = 0.092

#### Refinement

*R*[*F* ^2^ > 2σ(*F* ^2^)] = 0.048
*wR*(*F* ^2^) = 0.139
*S* = 1.07
5459 reflections
226 parameters
H-atom parameters constrained
Δρ_max_ = 0.43 e Å^−3^
Δρ_min_ = −0.24 e Å^−3^

### 

Data collection: *SMART* (Bruker, 2006 ▶); cell refinement: *SAINT* (Bruker, 2006 ▶); data reduction: *SAINT*; program(s) used to solve structure: *SIR97* (Altomare *et al.*, 1999 ▶); program(s) used to refine structure: *SHELXL97* (Sheldrick, 2008 ▶); molecular graphics: *PLATON* (Spek, 2009 ▶); software used to prepare material for publication: *PLATON*.

## Supplementary Material

Crystal structure: contains datablock(s) I, global. DOI: 10.1107/S1600536812004096/bt5808sup1.cif

Structure factors: contains datablock(s) I. DOI: 10.1107/S1600536812004096/bt5808Isup2.hkl

Supplementary material file. DOI: 10.1107/S1600536812004096/bt5808Isup3.cml

Additional supplementary materials:  crystallographic information; 3D view; checkCIF report

## Figures and Tables

**Table 1:** Hydrogen-bond geometry (Å, °)

*D*—H⋯*A*	*D*—H	H⋯*A*	*D*⋯*A*	*D*—H⋯*A*
N25—H25*A*⋯N15^i^	0.85	2.18	2.9866 (13)	158
N25—H25*B*⋯F24^ii^	0.86	2.44	3.0631 (11)	130

Symmetry codes: (i) 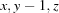; (ii) 
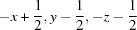
.

**Fig. 1.**
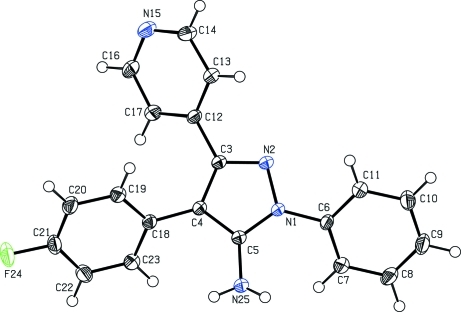
View of the title compound. Displacement ellipsoids are drawn at the 50% probability level. H atoms are depicted as circles of arbitrary size.

## supplementary crystallographic information

### Comment

Compounds having a vicinal 4-fluorophenyl/pyridin-4-yl system connected to a five-membered heterocyclic core have been considered to be potential p38α MAP kinase inhibitors (Abu Thaher *et al.* 2009, Peifer *et al.* 2006). Recently, we showed that the regioisomeric switch from 3-(4-fluorophenyl)-4-(pyridin-4-yl)-1-(aryl)-1*H*-pyrazol-5-amine to 4-(4-fluorophenyl)-3-(pyridin-4-yl)-1-(aryl)-1*H*-pyrazol-5-amine completely changed the inhibitory profile from p38α MAP kinase to kinases releant in cancer (Abu Thaher *et al.* 2012).

In the crystal structure of the title compound (Fig. 1), the pyrazole ring forms dihedral angels of 43.51 (6)°, 39.95 (6)° and 32.23 (6)° with the 4-fluorophenyl, pyridine and phenyl rings, respectively. The 4-fluorophenyl ring encloses dihedral angels of 46.49 (6)° and 17.4 (6)° toward the pyridine and phenyl rings, respectively. The pyridine ring is orientated at a dihedral angle of 30.07 (6)° toward the phenyl ring.

The crystal packing shows that the amino function acts as a hydrogen bond donor of two intermolecular hydrogen bonds - one to the nitrogen atom (N15) of the pyridine ring and another one to the fluorine atom (F24) of the 4-fluorophenyl ring of two different molecules. The length of the hydrogen bonds is 2.18 Å and 2.44 Å, respectively.

### Experimental

LDA (20 mmol) was added to 30 ml dry THF and cooled to 195 K. 4-Fluorophenyl acetonitrile (14 mmol) in 10 ml THF was added dropwise and the reaction was stirred for 45 min. *N*-Phenyl-4-pyridinecarbohydrazonoyl chloride (5 mmol) dissolved in THF was added slowly to the reaction and stirring was continued for 1 h. After warming to 293 K, water (50 ml) was added and the reaction mixture was extracted with ethyl acetate (2x 50 ml). The organic layer was dried over Na_2_SO_4_, concentrated to about 5 ml and the pure product precipitated as a pale brown solid, filtered and washed with petroleum ether. Yield: 30%. Crystals suitable for X-Ray were obtained from recrystallization from hot methanol.

### Refinement

Hydrogen atoms attached to carbons were placed at calculated positions with C—H = 0.95 Å (aromatic) or 0.98–0.99 Å (*sp*^3^ C-atom). All H atoms were refined in the riding-model approximation with isotropic displacement parameters set at 1.2–1.5 times of the *U*_eq_ of the parent atom. H atoms bonded to N were found in a difference map and constrained to this position with U(H)=1.5U_eq_(N).

### Crystal data

**Table:**

C_20_H_15_FN_4_	*F*(000) = 688
*M**_r_* = 330.36	*D*_x_ = 1.359 Mg m^−^^3^
Monoclinic, *P*2_1_/*n*	Mo *K*α radiation, λ = 0.71073 Å
Hall symbol: -P 2yn	Cell parameters from 8198 reflections
*a* = 12.2408 (3) Å	θ = 2.5–31°
*b* = 10.4427 (2) Å	µ = 0.09 mm^−^^1^
*c* = 12.9099 (3) Å	*T* = 193 K
β = 101.951 (1)°	Plate, colourless
*V* = 1614.46 (6) Å^3^	0.39 × 0.38 × 0.24 mm
*Z* = 4	

### Data collection

**Table:**

Bruker SMART CCD diffractometer	3972 reflections with *I* > 2σ(*I*)
Radiation source: sealed Tube	*R*_int_ = 0.092
Graphite monochromator	θ_max_ = 31.7°, θ_min_ = 2.1°
CCD scan	*h* = −17→18
29688 measured reflections	*k* = −15→15
5459 independent reflections	*l* = −19→19

### Refinement

**Table:**

Refinement on *F*^2^	Primary atom site location: structure-invariant direct methods
Least-squares matrix: full	Secondary atom site location: difference Fourier map
*R*[*F*^2^ > 2σ(*F*^2^)] = 0.048	Hydrogen site location: inferred from neighbouring sites
*wR*(*F*^2^) = 0.139	H-atom parameters constrained
*S* = 1.07	*w* = 1/[σ^2^(*F*_o_^2^) + (0.0759*P*)^2^] where *P* = (*F*_o_^2^ + 2*F*_c_^2^)/3
5459 reflections	(Δ/σ)_max_ = 0.001
226 parameters	Δρ_max_ = 0.43 e Å^−^^3^
0 restraints	Δρ_min_ = −0.24 e Å^−^^3^

### Special details

**Table:**

Geometry. All e.s.d.'s (except the e.s.d. in the dihedral angle between two l.s. planes) are estimated using the full covariance matrix. The cell e.s.d.'s are taken into account individually in the estimation of e.s.d.'s in distances, angles and torsion angles; correlations between e.s.d.'s in cell parameters are only used when they are defined by crystal symmetry. An approximate (isotropic) treatment of cell e.s.d.'s is used for estimating e.s.d.'s involving l.s. planes.
Refinement. Refinement of *F*^2^ against ALL reflections. The weighted *R*-factor *wR* and goodness of fit *S* are based on *F*^2^, conventional *R*-factors *R* are based on *F*, with *F* set to zero for negative *F*^2^. The threshold expression of *F*^2^ > σ(*F*^2^) is used only for calculating *R*-factors(gt) *etc*. and is not relevant to the choice of reflections for refinement. *R*-factors based on *F*^2^ are statistically about twice as large as those based on *F*, and *R*- factors based on ALL data will be even larger.

### Fractional atomic coordinates and isotropic or equivalent isotropic displacement parameters (Å^2^)

**Table:**

	*x*	*y*	*z*	*U*_iso_*/*U*_eq_	
N1	0.42742 (8)	0.43838 (8)	0.21818 (7)	0.01963 (19)	
N2	0.42901 (8)	0.56672 (8)	0.24356 (7)	0.0203 (2)	
C3	0.38505 (9)	0.62463 (9)	0.15232 (8)	0.0186 (2)	
C4	0.35487 (9)	0.53683 (9)	0.06717 (8)	0.0188 (2)	
C5	0.38251 (9)	0.41737 (9)	0.11336 (8)	0.0196 (2)	
C6	0.47171 (9)	0.34914 (9)	0.29983 (8)	0.0200 (2)	
C7	0.52687 (9)	0.23961 (10)	0.27749 (9)	0.0253 (2)	
H7	0.5349	0.2224	0.2072	0.030*	
C8	0.57028 (11)	0.15549 (11)	0.35910 (10)	0.0310 (3)	
H8	0.6058	0.0789	0.3440	0.037*	
C9	0.56218 (11)	0.18236 (11)	0.46214 (10)	0.0323 (3)	
H9	0.5923	0.1245	0.5175	0.039*	
C10	0.50995 (10)	0.29421 (12)	0.48467 (9)	0.0290 (3)	
H10	0.5067	0.3143	0.5557	0.035*	
C11	0.46249 (10)	0.37644 (10)	0.40313 (9)	0.0243 (2)	
H11	0.4239	0.4510	0.4179	0.029*	
C12	0.37406 (8)	0.76535 (9)	0.15138 (8)	0.0193 (2)	
C13	0.34059 (10)	0.82967 (10)	0.23403 (9)	0.0235 (2)	
H13	0.3249	0.7833	0.2926	0.028*	
C14	0.33030 (10)	0.96196 (10)	0.23020 (10)	0.0276 (3)	
H14	0.3088	1.0042	0.2880	0.033*	
N15	0.34895 (9)	1.03314 (9)	0.14963 (8)	0.0298 (2)	
C16	0.38196 (11)	0.97073 (11)	0.07086 (10)	0.0299 (3)	
H16	0.3964	1.0196	0.0131	0.036*	
C17	0.39641 (10)	0.83907 (10)	0.06831 (9)	0.0258 (2)	
H17	0.4211	0.7999	0.0108	0.031*	
C18	0.30291 (9)	0.56329 (9)	−0.04437 (8)	0.0193 (2)	
C19	0.21579 (9)	0.65213 (10)	−0.06991 (9)	0.0238 (2)	
H19	0.1874	0.6914	−0.0146	0.029*	
C20	0.17003 (10)	0.68406 (11)	−0.17433 (9)	0.0273 (3)	
H20	0.1119	0.7456	−0.1910	0.033*	
C21	0.21154 (10)	0.62388 (11)	−0.25283 (9)	0.0276 (3)	
C22	0.29435 (10)	0.53254 (11)	−0.23223 (9)	0.0269 (2)	
H22	0.3194	0.4908	−0.2884	0.032*	
C23	0.34046 (9)	0.50288 (10)	−0.12725 (9)	0.0229 (2)	
H23	0.3982	0.4408	−0.1116	0.027*	
F24	0.16930 (7)	0.65754 (7)	−0.35554 (6)	0.0425 (2)	
N25	0.37049 (9)	0.29779 (8)	0.06845 (7)	0.0264 (2)	
H25A	0.3602	0.2335	0.1059	0.040*	
H25B	0.3307	0.3008	0.0053	0.040*	

### Atomic displacement parameters (Å^2^)

**Table:**

	*U*^11^	*U*^22^	*U*^33^	*U*^12^	*U*^13^	*U*^23^
N1	0.0270 (5)	0.0128 (4)	0.0171 (4)	0.0019 (3)	−0.0001 (3)	−0.0001 (3)
N2	0.0273 (5)	0.0125 (4)	0.0195 (4)	0.0005 (3)	0.0010 (3)	−0.0006 (3)
C3	0.0216 (5)	0.0137 (4)	0.0197 (5)	0.0004 (4)	0.0024 (4)	−0.0001 (3)
C4	0.0219 (5)	0.0147 (4)	0.0184 (5)	0.0008 (4)	0.0010 (4)	0.0006 (3)
C5	0.0238 (5)	0.0150 (4)	0.0186 (5)	0.0005 (4)	0.0014 (4)	−0.0006 (3)
C6	0.0232 (5)	0.0149 (4)	0.0195 (5)	0.0000 (4)	−0.0008 (4)	0.0032 (3)
C7	0.0293 (6)	0.0213 (5)	0.0230 (5)	0.0043 (4)	0.0002 (4)	0.0003 (4)
C8	0.0342 (6)	0.0212 (5)	0.0335 (7)	0.0082 (5)	−0.0024 (5)	0.0023 (4)
C9	0.0372 (7)	0.0264 (6)	0.0282 (6)	0.0021 (5)	−0.0047 (5)	0.0098 (5)
C10	0.0357 (6)	0.0289 (6)	0.0205 (5)	−0.0018 (5)	0.0016 (5)	0.0048 (4)
C11	0.0298 (6)	0.0203 (5)	0.0218 (5)	0.0016 (4)	0.0035 (4)	0.0013 (4)
C12	0.0206 (5)	0.0135 (4)	0.0216 (5)	0.0005 (4)	−0.0008 (4)	0.0000 (3)
C13	0.0293 (6)	0.0169 (4)	0.0234 (5)	0.0018 (4)	0.0038 (4)	0.0008 (4)
C14	0.0329 (6)	0.0178 (5)	0.0314 (6)	0.0025 (4)	0.0054 (5)	−0.0033 (4)
N15	0.0364 (6)	0.0164 (4)	0.0342 (6)	0.0003 (4)	0.0018 (4)	0.0003 (4)
C16	0.0412 (7)	0.0171 (5)	0.0303 (6)	−0.0028 (5)	0.0047 (5)	0.0056 (4)
C17	0.0360 (6)	0.0181 (5)	0.0232 (6)	−0.0016 (4)	0.0063 (5)	0.0003 (4)
C18	0.0214 (5)	0.0152 (4)	0.0196 (5)	−0.0018 (4)	0.0002 (4)	0.0008 (3)
C19	0.0253 (5)	0.0203 (5)	0.0235 (5)	0.0024 (4)	0.0002 (4)	−0.0003 (4)
C20	0.0276 (6)	0.0222 (5)	0.0276 (6)	0.0018 (4)	−0.0044 (5)	0.0035 (4)
C21	0.0331 (6)	0.0269 (5)	0.0187 (5)	−0.0057 (5)	−0.0044 (4)	0.0045 (4)
C22	0.0326 (6)	0.0280 (5)	0.0196 (5)	−0.0029 (5)	0.0042 (4)	−0.0011 (4)
C23	0.0244 (5)	0.0203 (5)	0.0230 (5)	0.0010 (4)	0.0031 (4)	0.0003 (4)
F24	0.0545 (5)	0.0467 (5)	0.0197 (4)	−0.0003 (4)	−0.0078 (3)	0.0087 (3)
N25	0.0409 (6)	0.0144 (4)	0.0202 (5)	−0.0002 (4)	−0.0025 (4)	−0.0008 (3)

### Geometric parameters (Å, º)

**Table:**

N1—C5	1.3687 (13)	C13—C14	1.3871 (14)
N1—N2	1.3789 (11)	C13—H13	0.9500
N1—C6	1.4272 (13)	C14—N15	1.3360 (16)
N2—C3	1.3342 (13)	C14—H14	0.9500
C3—C4	1.4202 (13)	N15—C16	1.3389 (17)
C3—C12	1.4755 (13)	C16—C17	1.3875 (15)
C4—C5	1.3938 (13)	C16—H16	0.9500
C4—C18	1.4748 (13)	C17—H17	0.9500
C5—N25	1.3718 (12)	C18—C23	1.3992 (15)
C6—C7	1.3885 (15)	C18—C19	1.4004 (14)
C6—C11	1.3910 (16)	C19—C20	1.3891 (15)
C7—C8	1.3900 (15)	C19—H19	0.9500
C7—H7	0.9500	C20—C21	1.3760 (17)
C8—C9	1.3830 (19)	C20—H20	0.9500
C8—H8	0.9500	C21—F24	1.3658 (12)
C9—C10	1.3909 (17)	C21—C22	1.3771 (17)
C9—H9	0.9500	C22—C23	1.3909 (15)
C10—C11	1.3891 (15)	C22—H22	0.9500
C10—H10	0.9500	C23—H23	0.9500
C11—H11	0.9500	N25—H25A	0.8520
C12—C13	1.3924 (15)	N25—H25B	0.8591
C12—C17	1.3925 (15)		
			
C5—N1—N2	111.95 (8)	C14—C13—C12	119.48 (11)
C5—N1—C6	129.72 (8)	C14—C13—H13	120.3
N2—N1—C6	118.33 (8)	C12—C13—H13	120.3
C3—N2—N1	104.32 (8)	N15—C14—C13	123.58 (11)
N2—C3—C4	112.55 (9)	N15—C14—H14	118.2
N2—C3—C12	118.56 (9)	C13—C14—H14	118.2
C4—C3—C12	128.89 (9)	C14—N15—C16	116.59 (10)
C5—C4—C3	104.21 (9)	N15—C16—C17	124.09 (11)
C5—C4—C18	127.07 (9)	N15—C16—H16	118.0
C3—C4—C18	128.70 (9)	C17—C16—H16	118.0
N1—C5—N25	123.35 (9)	C16—C17—C12	118.90 (11)
N1—C5—C4	106.96 (8)	C16—C17—H17	120.6
N25—C5—C4	129.69 (10)	C12—C17—H17	120.6
C7—C6—C11	120.49 (10)	C23—C18—C19	118.25 (10)
C7—C6—N1	120.74 (10)	C23—C18—C4	121.24 (9)
C11—C6—N1	118.72 (9)	C19—C18—C4	120.50 (10)
C6—C7—C8	119.29 (11)	C20—C19—C18	121.46 (11)
C6—C7—H7	120.4	C20—C19—H19	119.3
C8—C7—H7	120.4	C18—C19—H19	119.3
C9—C8—C7	120.56 (11)	C21—C20—C19	117.96 (10)
C9—C8—H8	119.7	C21—C20—H20	121.0
C7—C8—H8	119.7	C19—C20—H20	121.0
C8—C9—C10	119.94 (10)	F24—C21—C20	118.31 (11)
C8—C9—H9	120.0	F24—C21—C22	118.77 (11)
C10—C9—H9	120.0	C20—C21—C22	122.92 (10)
C11—C10—C9	119.93 (11)	C21—C22—C23	118.44 (11)
C11—C10—H10	120.0	C21—C22—H22	120.8
C9—C10—H10	120.0	C23—C22—H22	120.8
C10—C11—C6	119.71 (11)	C22—C23—C18	120.90 (10)
C10—C11—H11	120.1	C22—C23—H23	119.6
C6—C11—H11	120.1	C18—C23—H23	119.6
C13—C12—C17	117.35 (9)	C5—N25—H25A	119.6
C13—C12—C3	121.00 (9)	C5—N25—H25B	111.0
C17—C12—C3	121.66 (10)	H25A—N25—H25B	116.5
			
C5—N1—N2—C3	−0.46 (12)	N1—C6—C11—C10	−176.75 (10)
C6—N1—N2—C3	178.81 (9)	N2—C3—C12—C13	−40.18 (15)
N1—N2—C3—C4	−0.21 (12)	C4—C3—C12—C13	139.92 (12)
N1—N2—C3—C12	179.88 (9)	N2—C3—C12—C17	139.91 (11)
N2—C3—C4—C5	0.76 (13)	C4—C3—C12—C17	−39.99 (17)
C12—C3—C4—C5	−179.34 (11)	C17—C12—C13—C14	0.40 (16)
N2—C3—C4—C18	179.05 (10)	C3—C12—C13—C14	−179.51 (10)
C12—C3—C4—C18	−1.05 (19)	C12—C13—C14—N15	1.25 (18)
N2—N1—C5—N25	−179.34 (10)	C13—C14—N15—C16	−1.73 (18)
C6—N1—C5—N25	1.49 (18)	C14—N15—C16—C17	0.61 (18)
N2—N1—C5—C4	0.95 (13)	N15—C16—C17—C12	0.97 (19)
C6—N1—C5—C4	−178.22 (11)	C13—C12—C17—C16	−1.43 (16)
C3—C4—C5—N1	−0.99 (12)	C3—C12—C17—C16	178.48 (10)
C18—C4—C5—N1	−179.31 (10)	C5—C4—C18—C23	−45.51 (16)
C3—C4—C5—N25	179.33 (12)	C3—C4—C18—C23	136.58 (12)
C18—C4—C5—N25	1.0 (2)	C5—C4—C18—C19	135.85 (12)
C5—N1—C6—C7	33.57 (17)	C3—C4—C18—C19	−42.06 (16)
N2—N1—C6—C7	−145.55 (10)	C23—C18—C19—C20	−2.48 (16)
C5—N1—C6—C11	−148.95 (11)	C4—C18—C19—C20	176.19 (10)
N2—N1—C6—C11	31.93 (15)	C18—C19—C20—C21	1.16 (17)
C11—C6—C7—C8	1.73 (17)	C19—C20—C21—F24	−178.07 (10)
N1—C6—C7—C8	179.17 (10)	C19—C20—C21—C22	1.21 (18)
C6—C7—C8—C9	−2.19 (18)	F24—C21—C22—C23	177.17 (10)
C7—C8—C9—C10	0.18 (19)	C20—C21—C22—C23	−2.11 (18)
C8—C9—C10—C11	2.31 (19)	C21—C22—C23—C18	0.67 (17)
C9—C10—C11—C6	−2.76 (18)	C19—C18—C23—C22	1.54 (16)
C7—C6—C11—C10	0.74 (17)	C4—C18—C23—C22	−177.13 (10)

### Hydrogen-bond geometry (Å, º)

**Table:**

*D*—H···*A*	*D*—H	H···*A*	*D*···*A*	*D*—H···*A*
N25—H25*A*···N15^i^	0.85	2.18	2.9866 (13)	158
N25—H25*B*···F24^ii^	0.86	2.44	3.0631 (11)	130

Symmetry codes: (i) *x*, *y*−1, *z*; (ii) −*x*+1/2, *y*−1/2, −*z*−1/2.
